# Association analysis of single-nucleotide polymorphism in prolactin and its receptor with productive and body conformation traits in Liaoning cashmere goats

**DOI:** 10.5194/aab-65-145-2022

**Published:** 2022-04-21

**Authors:** Yanzhi Wu, Yu Zhang, Yuting Qin, Weidong Cai, Xinjiang Zhang, Yanan Xu, Xingtang Dou, Zhanhong Wang, Di Han, Jiaming Wang, Guangyu Lin, Lingling Wang, Jianjun Hao, Shuqing Fu, Rui Chen, Yinggang Sun, Zhixian Bai, Ming Gu, Zeying Wang

**Affiliations:** 1 College of Animal Science & Veterinary Medicine, Shenyang Agricultural University, Shenyang 110866, China; 2 Liaoning Province Modern Agricultural Production Base Construction Engineering Center, Liaoyang 110000, China; 3 Administration Bureau of Zhungeer Banner, Ordos City, Inner Mongolia 010399, China; 4 Lantian Sub-district Office, Zhungeer Banner, Ordos City, Inner Mongolia 010399, China

## Abstract

The results of this study showed that the single-nucleotide polymorphism (SNP) sites of the *PRL* and *PRLR* genes have a certain association with the milk production performance, body size and cashmere performance of Liaoning cashmere goats (LCGs). Through our designed experiment, the potential SNPs of LCG were detected by sequence alignment, and two SNPs were found on two genes. The CC genotype of the *PRL* gene is the dominant genotype among the three genotypes. The GG genotype of the *PRLR* gene is the dominant genotype among the two genotypes. At the same time, the two genotypes also have good performance in cashmere production and body size. Through the screening of haplotype combination, the milk fat rate > 7.6 %, the milk protein rate > 5.6 %, the milk somatic cell number < 1500 × 10 ${}^{3}$ mL ${}^{-1}$ , the cashmere fineness < 15.75 µ m, the chest girth > 105 cm, the chest depth > 33 cm, and the waist height > 67.5 cm are considered as screening indexes for comprehensive production performance of Liaoning cashmere goats. It is concluded that the GCGC type is the dominant haplotype combination. According to our research data, we found that the biological indicators of Liaoning cashmere goat milk are higher than the national standards, so we think it is very significant to study the milk production performance of our experiment. Further research can be done on goat milk production and body conformation traits around *PRL* gene and *PRLR* gene.

## 1 Introduction

Liaoning cashmere goat is an important breed in China. It shows the highest production of cashmere in China and even in the world. The milk of Liaoning cashmere goat is rich of nutrients, with the fat, protein, lactose and somatic cell contents of 3.58 %, 2.58 %, 2.14 % and $187\times 10^{3}$ mL ${}^{-1}$ , respectively (Stergiadis et al., 2019). The goat milk-based formula reveals similar growth and nutritional outcomes in infants to standard whey-dominant cow milk-based formula (Zhou et al., 2014; Xu et al., 2015), and the content of bioavailable N in goat milk-based infant formula is similar to human milk (Maathuis et al., 2017). Lamb is a popular meat because of its high protein, low fat and cholesterol properties (Fernandes et al., 2014). Body size is an indicator of meat production ability in mammals. Thus, in addition to cashmere production, milk production performance and body size of Liaoning cashmere goats are also of great economic significance.

Prolactin (PRL) is a protein hormone mainly secreted by the eosinophils in anterior pituitary gland and regulating lactation and reproductive functions in animals (Li et al., 2017). During the development of animal mammary glands, prolactin plays a key role, regulating the differentiation and proliferation of mammary gland cells to promote milk production (Uddin et al., 2013). Moreover, PRL plays important roles in the synthesis of lactose, as well as regulation of protein and fat contents in milk (Dahl, 2008; Freeman et al., 2000). Previous studies indicated that the prolactin receptor (*PRLR*) is an important candidate gene affecting the reproductive traits of livestock and poultry, including pig, sheep, chicken and geese (Liu et al., 2020). A high correlation between *PRLR* and lactation performance was revealed in dairy cows (Verardo et al., 2021; Leyva-Corona et al., 2018). At the molecular level, it has been demonstrated that oxytocin induced lactation through PRLR dimerization (Lü et al., 2010). After binding to PRL, PRLP activates JAK2 kinase and phosphorylate the STAT5 transcription factor to exert functions (Bolefeysot et al., 1998; Dahl, 2008; Uddin et al., 2013). The intracellular domain of PRLR is highly disordered (Haxholm et al., 2015) and shows a high dynamic at the second or third protein structure levels. Thus, PRLP cannot form a stable three-dimensional structure (Uversky, 2017; Paré et al., 2021). In goats, two internal variants of *PRLR* have been reported to associate with the growth performance (Liu et al., 2020). Due to the high conservation of *PRL* and *PRLR* among species in the same class (Xu et al., 2011; Ding, 2009), *PRL* and *PRLR* are assumed to regulate growth and milk protein in Liaoning cashmere goat.

Single-nucleotide polymorphism (SNP) is a widely used genetic indicator associated with functional phenotypes. Up to date, the variation of SNPs in the *PRL* and *PRLP* have not been reported in Liaoning cashmere goat, and their effects on growth and milk production remain unknown. In the present study, the *PRL* and *PRLR* genes were sequenced in 1177 Liaoning cashmere goats, and the SNP loci were identified. In addition, the association between growth/milk indices and SNP variation were analyzed. These results might facilitate the understanding of biological functions of PRL-PRLR pathway in goat and contribute to further molecular breeding of goat using the SNP loci in *PRL* and the *PRLR* as the potential biomarkers.

## 2 Materials and methods

### 2.1 Experimental animals and sample collection

The present study was approved by the Experimental Animal Management Committee of Shenyang Agricultural University, and all the experimental procedures carefully followed the Regulations of the Standing Committee of the People's Congress of Liaoning Province.

A total of 1177 Liaoning cashmere goats were used in the present study, which were selected from the Construction Engineering Center of Modern Agricultural Production Base in Liaoning Province, China. They were raised on the same farm. All goats were 3-year-old ewes and in good health. Their body weights were 54.74±3.48 kg (mean ± SD), with the maximum and minimal values of 50.03 and 59.73 kg, respectively. From each animal, 5 mL of venous blood was collected from the jugular vein at 08:00–10:00 UTC + 8 in East District 8. by a professional veterinarian for extraction of genomic DNA. The blood samples were anticoagulated using EDTA and then preserved at -20 ${}^{\circ }$ C.

Cashmere production performance was uniformly determined by the professional and technical personnel from the Liaoning Academy of Animal Husbandry. The body size data were measured according to the test method mentioned in *Livestock Breeding* published by China Agriculture Press (Zhang, 2001). The milk production performance data were measured by the Liaoning Province Production Performance Testing Center. FUNKE GEBER LactoStar Automatic Milk Analyzer and LACTOSCAN MCC COMBO Milk Analyzer Somatic Cell Counter were used to determine milk performance data.

### 2.2 DNA extraction

In this experiment, the EZ-10 Spin Column Blood Genomic DNA Purification Kit produced by the Shanghai Bioengineering Co. Ltd. (Shanghai, China) was used to extract the genomic DNA according to the manufacture's protocol, and the DNA samples were stored at -20 ${}^{\circ }$ C.

### 2.3 Primer design

Since the goat *PRL* gene sequence has not been reported, we used the full-length sequences of cattle *PRL* gene and goat *PRLR* gene in the National Center for Biotechnology Information (NCBI) to design specific primers for the coding regions using the Primer Premier 5.0 (Table A1 in Appendix A). The primers were synthesized by the Shanghai Bioengineering Co., Ltd. (Shanghai, China).

### 2.4 PCR amplification, sequencing and SNP analyses

The PCR reaction was carried out in 50 µ L of system, including 25 µ L of 2X SanTaq PCR Mix premix, 1 µ L of DNA template, 2 µ L of forward primer, 2 µ L of reverse primer, and 20 µ L of sterilized ddH ${}_{2}$ O. The PCR program included pre-denaturation at 94 ${}^{\circ }$ C for 3 min, 30 cycles of denaturation at 94 ${}^{\circ }$ C for 30 s, annealing at 50 ${}^{\circ }$ C for 30 s, and extension at 72 ${}^{\circ }$ C for 60 s, as well as final extension at 72 ${}^{\circ }$ C for 10 min. The PCR products were evaluated by electrophoresis on 1 % agarose gel. The PCR products were sequenced by the Shanghai Bioengineering Co., Ltd. (Shanghai, China), and the SNP loci were analyzed by alignment of all sequences.

## 3 Statistical analysis

The associations between the lactation, body size, cashmere production indices, and the SNPs of *PRL* and *PRLR* genes were evaluated by the one-way ANOVA analysis or independent Student's t test using the SPSS Statistics 20 (Park et al., 2009). The genotype frequency, allele frequency, $\chi^{2}$ value, polymorphism information content (PIC), effective number of alleles (Ne) and heterozygosity (He) were statistically analyzed. The completeness of the animal model was analyzed using the following equation.

$$Y_{ijkl}=\mu +h_{i}+p_{j}+s_{k}+m_{l}+e_{ijkl},$$

where $Y_{ijkl}$ is the observed value, μ is the overall average, $h_{i}$ is the influence of genotype or combined haploid, $p_{j}$ is the influence of season and farm, $s_{k}$ is the influence of year, $m_{l}$ is the influence of paternal decline, and $e_{ijkl}$ is the random error. The Duncan method was used for multiple comparisons. Different lowercase and uppercase letters in the superscripts mean significant difference at the 0.05 and 0.01 levels, respectively.

## 4 Results

### 4.1 SNP map in the *PRL* and *PRLR* genes

Alignments of all *PRL* and *PRLR* genes sequences revealed one SNP site (C442T) in the *PRL* gene (Fig. A1 in Appendix A) and one SNP site (G168061A) in the *PRLR* gene (Fig. A2).

### 4.2 Population genetic analysis

Based on the statistical analysis of the SNP sites (Tables A3, A4), *PRL* gene showed two alleles (U, u) and three genotypes (UU, Uu, uu). In comparison, *PRLR* gene had two allele (U, u) but formed only two genotypes (UU, Uu). The genotype frequency and allele frequency of the two mutation sites were different, suggesting that the expression products of these two genes might unevenly distribute in the Liaoning cashmere goat population. Among the tested animals, CT and GG genotypes (on the *PRL* and *PRLR* genes, respectively) showed the highest frequency and were the dominant genotypes; C and G alleles displayed the highest frequency and were considered dominant alleles, respectively. Polymorphism information content (PIC), expected heterozygosity (He) and effective allele (Ne) are three indices representing the degree of genetic variation in populations. The PIC value of the mutation site of C442T was greater than 0.25 but less than 0.5, indicating that it was moderately polymorphic. He and Ne are slightly higher, suggesting a moderate genetic heterozygosity and a moderate ability to preserve alleles during mutation or genetic drift. The PIC value of the mutation site of G168061A was less than 0.25, revealing a low polymorphism. The He and Ne values on this site were also low, demonstrating low genetic heterozygosity and locus variation. The P value of the C442T mutation site was much larger than 0.05, and $\chi^{2}$ was less than 1, indicating that this site followed the Hardy–Weinberg equilibrium. Under natural selection, the gene and genotype frequencies of this site could be stably transmitted to offspring across generations. The P value of the G168061A mutation site was less than 0.05, and $\chi^{2}$ was greater than 1, suggesting that this site was not in the Hardy–Weinberg equilibrium. Artificial selection might change its gene and genotype frequencies.

### 4.3 Comparison of milk production performance between different genotypes at each SNP site

At the C442T site of the *PRL* gene, the TT and CC types showed significantly higher fat, protein, urea, N contents, TS and somatic cell count than the CT type; TT showed significantly lower lactose content than CT and CC types (Table A4). At the G168061A locus in the *PRLR* gene, the AG genotype showed significantly higher fat, and lactose contents but lower protein content than the GG type (Table A4).

### 4.4 Comparison of body metrics between different SNP types

For the C442T locus on the *PRL* gene, the TT and CC types showed significantly higher body height, sacral height, chest width, hip bone width, shin circumference and waist height than the CT type. For the G168061A locus on the *PRLR* gene, the GG type revealed significantly higher chest depth, width but lower hip bone width, chest girth, shin circumference and waist height than the AG type (Table A5).

### 4.5 Comparison of body cashmere indices between different SNP genotypes

Among the three genotypes on the C442T locus of the *PRL* gene, the TT genotype showed the best cashmere yield, shearing weight, cashmere fineness and cashmere length. The CT genotype revealed the best net cashmere rate and cashmere weight. For the G168061A locus on the *PRLR* gene, the GG genotype displayed better cashmere weight but lower cashmere fineness than the AG genotype (Table A6).

### 4.6 Gene substitution effect

The additive effect values of the C442T locus of *PRL* gene and the G168061A locus of *PRLR* gene were negative, indicating that the mutation of this gene might positively affect animal performance. The C442T mutation increased animal production performance by 0.290 %, and the G168061A mutation increased animal production performance by 106.166 %.

### 4.7 Haplotype combination of two SNP sites

The C442T and G168061A site had three and two genotypes, respectively. SHEsis (http://analysis.bio-x.cn/myAnalysis.php, last access: 14 April 2021) analysis identified six haplotype combinations in the 1043 Liaoning cashmere goats (Table A7). Taking the lactation performance as the major index for analysis, H1H1 : GCGC was the superiority haplotype combination. Its milk protein content, milk fat content, N content and SnF were relatively higher, and its milk somatic cell count and urea content were at the medium levels (Table A8). Taking body size as the major index for consideration, H1H1 : GCGC and H1H3 : GTGT were the dominant haplotypes, and their body height, sacral height and waist height are prominent (Table A9). Taking cashmere production performance as the main consideration index, H1H1 : GCGC is the dominant haplotype. This haplotype has finer villus fineness, high net cashmere rate and outstanding performance (Table A10).

### 4.8 Combined analysis of haplotype lactation performance and body size

Liaoning cashmere goats are unique local breeds, and there is no clear standard for evaluating the quality of lactation performance of Liaoning cashmere goats. Thus, this study is of great significance for the association analysis of lactation performance and other production performance of Liaoning cashmere goats. Based on Tables A8 and A9, we use milk fat rate > 7.6 %, milk protein rate > 5.6 % and milk somatic cell number < 1500 × 10 ${}^{3}$ mL ${}^{-1}$ as the lactation performance screening criteria, and then we use chest circumference > 105 cm, chest depth > 33 cm and waist height > 67.5 cm as body size screening criteria. The screening results showed that H1H1 : GCGC was the dominant haplotype.

### 4.9 Combined analysis of haplotype lactation performance and body cashmere performance

Based on Tables A8 and A10, we use milk fat rate > 7.6 %, milk protein rate > 5.6 % and milk somatic cell number < 1500 × 10 ${}^{3}$ mL ${}^{-1}$ as the lactation performance screening index, and then we use cashmere fineness < 15.75 µ m as the cashmere production performance screening index. The screening results showed that H1H1 : GCGC was the dominant haplotype.

### 4.10 Combined analysis of haplotype body size and body cashmere performance

Based on Tables A9 and A10, we used chest circumference > 105 cm, chest depth > 33 cm and waist height > 67.5 cm as the screening criteria for body size, and then we used cashmere fineness < 15.75 µ m as the screening index for cashmere production performance. The screening results showed that H1H1 : GCGC was the dominant haplotype.

## 5 Discussion

In recent years, SNP technology has been used in cashmere goat production performance analysis widely. Mutation of *LALBA* gene affects cashmere quality in Inner Mongolia cashmere goats (Lan et al., 2008). Different genotypes and haplotypes of *FecB* and *ESR* genes influence litter size in Liaoning cashmere goats (Chang et al., 2019). However, there are still a lot of research gaps on the body size and milk production of cashmere goats. The assistance of *PRL* gene and *PRLR* gene for cashmere goat breeding can also be discussed in depth. In goats, the polymorphism of *PRL* gene and *PRLR* gene has been investigated widely. Polymorphic loci have been found in the *PRL* gene and associated with cashmere performance in native Chinese goats (Lan et al., 2009). Guo Haiyan and Zhang Yuan investigated the *PRLR* genes of Hu sheep and Jining Qing goats, respectively, and found that the *PRLR* genes of these two goats are polymorphic, but there is little relationship with litter size (Guo et al., 2017; Zhang et al., 2020).

Goat milk is rich of protein and calcium (Turck, 2013). From the results of our study, the milk of Liaoning cashmere goats contains higher protein and fat, which have good economic value in production. In terms of lactation performance, many studies have shown that the variation of the *PRL* gene and *PRLR* genes is statistically related to the lactation ability of dairy cows, and the mutation of *PRL* and *PRLR* has a greater impact on milk production performance (Capparelli et al., 2008; Lü et al., 2010; Hallerman et al., 1988; Sasavage et al., 1982; Sirja et al., 2006; Lü et al., 2011; Fallin et al., 2001). Thus, it is not surprising that the *PRLR* and *PRL* genes are significantly correlated with the lactation performance of Liaoning cashmere goats. According to the $\chi^{2}$ test, the *PRL* gene has been relatively stable in the inheritance of generations, and it conforms to the Hardy–Weinberg law, indicating that the direction of natural selection coincides with the direction of artificial selection, and the population gene frequency will tend to stabilize. The *PRLR* gene is not stable enough and does not comply with the Hardy–Weinberg law, indicating that artificial selection and natural selection have a deviation, and it also proves that mutation of *PRLR* gene brings a superior effect, and the mutation will be retained as far as possible in the process of artificial selection.

This study takes milk production performance as the main production performance consideration index, body size and cashmere production performance as secondary indicators. In the genotyping analysis of the *PRL* gene and *PRLR* SNP loci, the milk protein content is the most important measurement index. The CC genotype in C442T locus and the GG genotype of G168061A has outstanding performance in lactation, cashmere production and body size, respectively. The average milk fat rate of the AG genotype of G168061A was highest, and the AG genotype of G168061A can be bred in the direction of the pursuit of flavor because it contains a very high milk fat ratio. On the basis of genotyping analysis, we also analyzed the haplotype combinations of Liaoning cashmere goats. Compared with genotype analysis, the information content of haplotype combinations is richer, the analysis content is more comprehensive, and the results are relatively more reliable (Capparelli et al., 2008; Fallin et al., 2001). In this study, two SNP loci of *PRL* gene and *PRLR* gene were involved in haplotype combinations. The results showed that H1H1 : GCGC is superiority in cashmere performance, body size and lactation performance. The performance of other haplotype combinations is not outstanding. PRL and PRLR play a role in promoting the growth of human hair follicles and are involved in regulating the development of many kinds of mammalian skin and hair follicles (Langan et al., 2010; Craven et al., 2001; Foitzik et al., 2003; Littlejohn et al., 2014). In addition, PRL and PRLR are involved in the process of bone metabolism. Abnormal levels of prolactin often lead to abnormal bone metabolism (Giustina et al., 2008). Individual growth levels are closely related to PRL and PRLR. The haplotype results in this experiment also pointed to the excellent performance of specific haplotypes in three production performances. We speculate that because *PRL* genes and *PRLR* genes have an important influence on basic physiological functions, production performance is related to vitality, while the vitality of disadvantaged individuals is relatively low. It is easy to be eliminated under the conditions of natural selection and artificial selection. The production performance we select also has a certain impact on animal viability. Therefore, the haplotype results screened by different production performance data are relatively consistent. It is very likely that the haplotype Liaoning cashmere goat has great production potential and promotes molecular breeding.

## 6 Conclusion

Our study confirmed that the CC genotype (C442T) of *PRL* and the GG genotype of *PRLR* had advantages in lactation performance and cashmere production, and the TT genotype of *PRL* had advantages in body size performance. The GCGC haplotype combination had outstanding performance in lactation, body size and cashmere production performance. Therefore, GCGC can be used as the dominant haplotype combination of *PRL* and *PRLR* genes. We consider that the CC genotype of the *PRL* gene and the GG genotype of the *PRLR* gene can be used as molecular markers for in-depth research in the future.

## Supplement

10.5194/aab-65-145-2022-supplement The supplement related to this article is available online at: https://doi.org/10.5194/aab-65-145-2022-supplement.

## Data Availability

The data sets used in this article can be requested from the corresponding author.

## Appendix

The supplement related to this article is available online at: https://doi.org/10.5194/aab-65-145-2022-supplement.
