# Supplementary material for: Association analysis of single-nucleotide polymorphism in prolactin and its receptor with productive and body conformation traits in Liaoning cashmere goats
Source: Arch Anim Breed. 2022 Apr 21;65(2):145–55. doi: 10.5194/aab-65-145-2022 (PMC9051658; doi:10.5194/aab-65-145-2022)
Supplement: The supplement related to this article is available online at: https://doi.org/10.5194/aab-65-145-2022-supplement. [file aab-65-145-supplement.zip › aab-65-145-2022-supplement-title-page.pdf]

Supplement of Arch. Anim. Breed., 65, 145–155, 2022
https://doi.org/10.5194/aab-65-145-2022-supplement
© Author(s) 2022. CC BY 4.0 License.

Open Access
Archives Animal Breeding

Supplement of

Association analysis of single-nucleotide polymorphism in prolactin and its receptor with productive and body conformation traits in Liaoning cashmere goats

Yanzhi Wu et al.

Correspondence to: Zeying Wang (wangzeying2012@syau.edu.cn)

- aab-65-145-2022-supplement-title-page.pdf
- PRL .jpg
- PRLR .jpg

The copyright of individual parts of the supplement might differ from the article licence.
