# Supplementary figures and images for: Association analysis of single-nucleotide polymorphism in prolactin and its receptor with productive and body conformation traits in Liaoning cashmere goats
Source: Arch Anim Breed. 2022 Apr 21;65(2):145–55. doi: 10.5194/aab-65-145-2022 (PMC9051658; doi:10.5194/aab-65-145-2022)

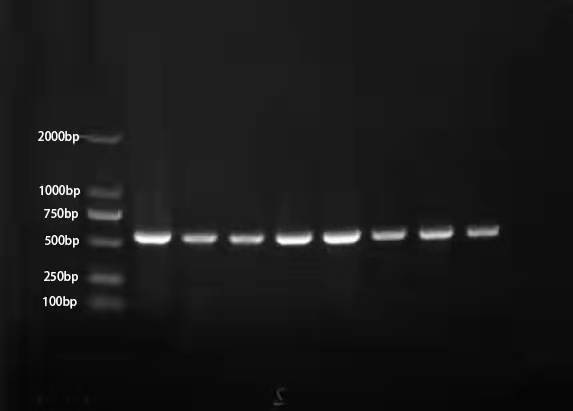

Supplement: The supplement related to this article is available online at: https://doi.org/10.5194/aab-65-145-2022-supplement. [file aab-65-145-supplement.zip › PRL .jpg]

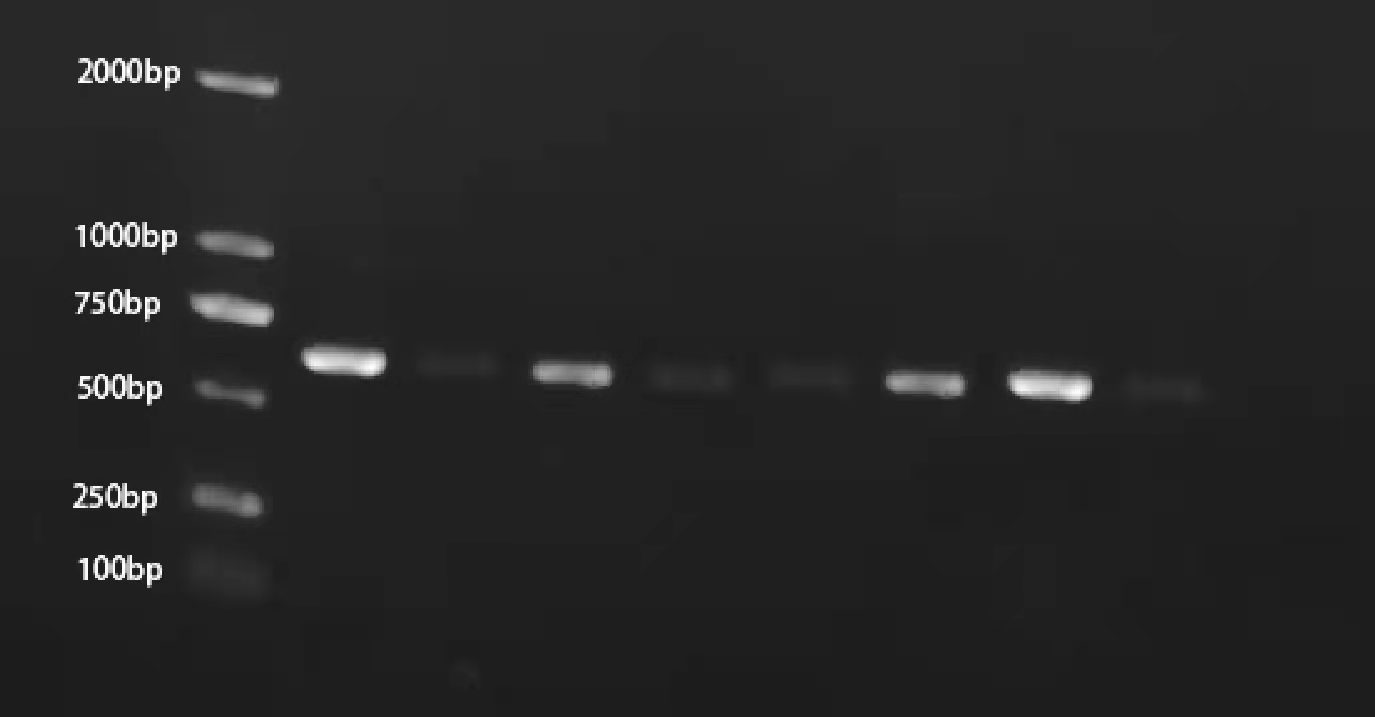

Supplement: The supplement related to this article is available online at: https://doi.org/10.5194/aab-65-145-2022-supplement. [file aab-65-145-supplement.zip › PRLR .jpg]
